# Cell therapies for immune-mediated disorders

**DOI:** 10.3389/fmed.2025.1550527

**Published:** 2025-03-26

**Authors:** Natalia Wiewiórska-Krata, Bartosz Foroncewicz, Krzysztof Mucha, Radosław Zagożdżon

**Affiliations:** ^1^Laboratory of Cellular and Genetic Therapies, Center for Preclinical Research, Medical University of Warsaw, Warsaw, Poland; ^2^ProMix Center (ProteogenOmix in Medicine), Department of Clinical Immunology, Medical University of Warsaw, Warsaw, Poland; ^3^Department of Transplantology, Immunology, Nephrology and Internal Diseases, Medical University of Warsaw, Warsaw, Poland; ^4^Institute of Biochemistry and Biophysics, Polish Academy of Sciences, Warsaw, Poland

**Keywords:** cell therapies, immune disorders, transplantation, regenerative medicine, Treg cells

## Abstract

Immune-mediated disorders are a broad range of diseases, arising as consequence of immune defects, exaggerated/misguided immune response or a mixture of both conditions. Their frequency is on a rise in the developed societies and they pose a significant challenge for diagnosis and treatment. Traditional pharmacological, monoclonal antibody-based or polyclonal antibody replacement-based therapies aiming at modulation of the immune responses give very often dissatisfactory results and/or are burdened with unacceptable adverse effects. In recent years, a new group of treatment modalities has emerged, utilizing cells as living drugs, especially with the use of the up-to-date genetic engineering. These modern cellular therapies are designed to offer a high potential for more targeted, safe, durable, and personalized treatment options. This work briefly reviews the latest advances in the treatment of immune-mediated disorders, mainly those related to exaggeration of the immune response, with such cellular therapies as hematopoietic stem cells (HSCs), mesenchymal stromal cells (MSCs), regulatory T cells (Tregs), chimeric antigen receptor (CAR) T cells and others. We highlight the main features of these therapies as new treatment options for taming the dysregulated immune system. Undoubtfully, in near future such therapies can provide lasting remissions in a range of immune-mediated disorders with reduced treatment burden and improved quality of life for the patients.

## 1 Introduction

Properly functioning immune system is indispensable for human health as it defends the body against pathogens, cancer and other foreign threats. However, it must be tamed by tolerance mechanisms to spare healthy tissues and co-exist with the commensal microbiome. The complex nature of immune system makes it vulnerable to malfunctions, either in a form of immune deficiencies, exaggerated or misdirected immune activation or a co-existence of both types of these pathological conditions. The consequences of these abnormalities are termed the immune-mediated disorders (IMD). Overall, IMD are currently affecting up to 10% of the population (1–3). In this review, we will mostly focus on the abnormal exaggeration of the immune responses, sometimes referred to as immune-mediated inflammatory disorders (IMID), as the most remarkable progress has been done in cellular therapies in this field (1).

**Figure 1:**
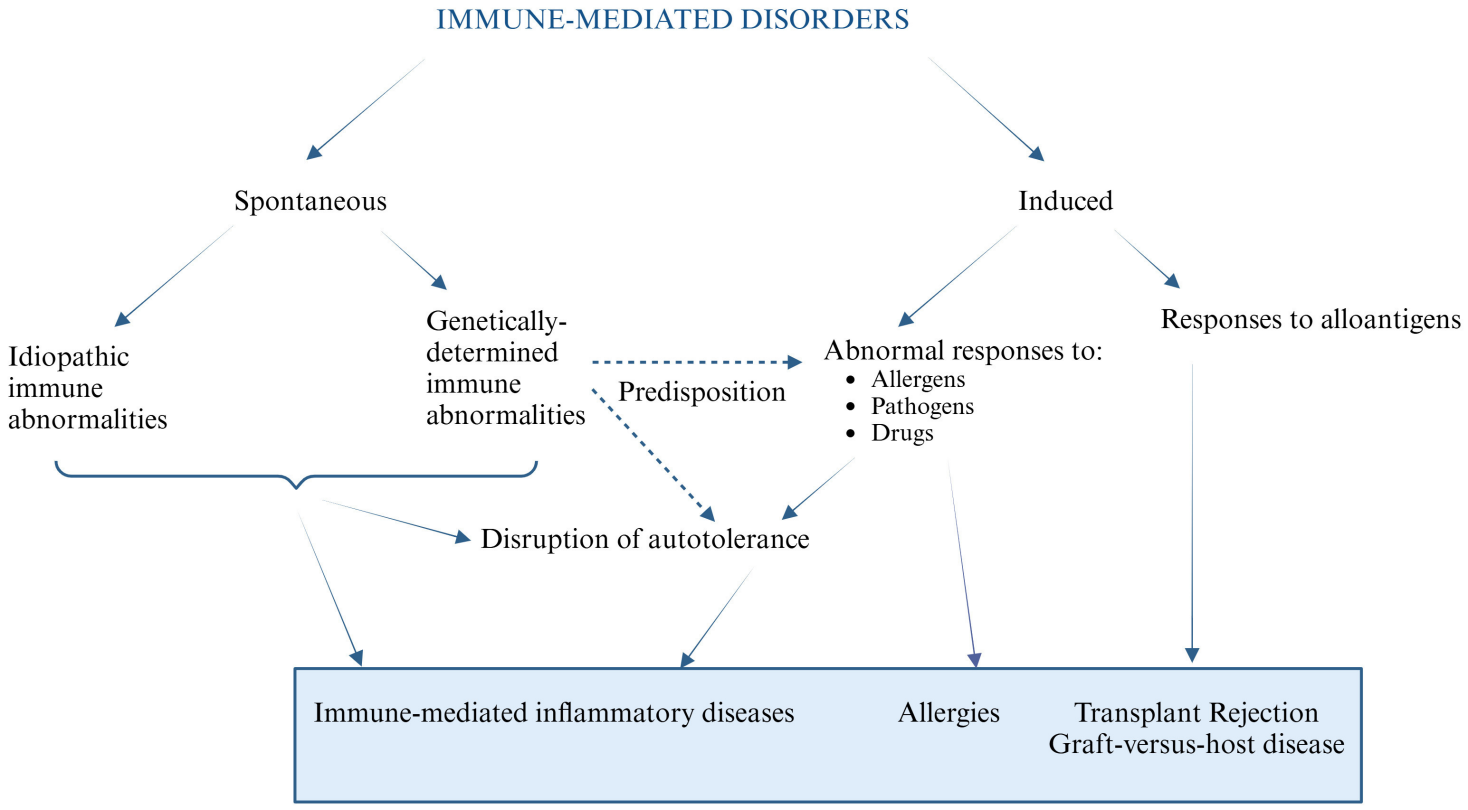
An overview of the pathomechanisms of immune mediated disorders.

It is important to mention that the overactivity of the immune system can be primary/spontaneous (idiopathic or caused by a pathological genetic trait) or induced as secondary to immune defects or external/environmental factors (e.g., allergens, pathogenic infections or pharmacological treatment), and can also occur under specific conditions of allogeneic organ transplantation (4–6). The disbalance of immune system can eventually cause damage to the inflamed tissues, leading to their functions impairment and diseases development. The main groups of such illnesses are autoinflammatory syndromes, allergies and rejection of the transplanted organ and other immune-mediated inflammatory diseases (Figure 1). Many of these conditions are chronic and require long-term management.

Importantly, the cross-relations between various IMD, indicate a potential gene-related common predispositions. Especially among connective tissue diseases, such as systemic lupus erythematosus (SLE) and rheumatoid arthritis, between diabetes, celiac disease, and thyroid disorders (2), as well as allergic reactions and inflammatory diseases (7). Indeed, variants of approximately 500 genes are currently suspected as capable of posing a predisposition to immune dysregulation (8), and this list is definitely still incomplete. Moreover, several clinical factors can significantly enhance or suppress the personal immune responses (9), resulting in modulation of the disease progression. Among the key clinical factors (Table 1), the most pronounced are: age, gender, pre-existing chronic diseases, infections, and therapies.

**Table 1:** Examples of clinical factors affecting the immune response and IMD development.

**History of diseases and infections**	**History of diseases and infections, treatment (chemotherapy, immunosuppression, immunomodulation)**.
Age	Young age (< 18 years): greater risk of disease occurrence, faster diagnosis, better prognosis and response to treatment; adult age (> 18 years): lower incidence of disease, complex diagnosis, poorer prognosis and limited response to treatment; elderly (>65 years): second peak of greater disease incidence, complex diagnosis, poorer prognosis and limited response to treatment.
Gender	Increased risk for IMD in women comparing to men, most probably as a result of endocrine (estrogen and testosterone) balance.
Extrinsic factors	Pre-existing immunity, dysbiosis, infections, antibiotics, geographic location, season, family size, exposition to toxins, smoking, alcohol consumption, exercising, sleep, nutrition, body mass index, micronutrients.
Physiological mechanisms	Skin, mucous membranes, microbiota, enzymes, physiological, reflexes, accelerated metabolism, phagocytosis.

Infections control is particularly important for IMD management. Infections can both stimulate the disease onset and exacerbate its course. Among infants and young children, the immune system develops, that might lead to a higher susceptibility to infections, while the elderly immune system, which naturally weakened with age, has a reduced effectiveness against infections (10). Other significant factors, such as malnutrition (11), obesity (12), chronic stress (13), sleep deprivation (14), exposure to pathogens or pollutants (15), lifestyle (low physical activity, smoking and alcohol consumption) (16–18), can also impair immune function and increase susceptibility to infections. These factors influence the immune system in complex ways and can make the results of applied therapies inadequate. Hence, understanding the interplay of these factors is crucial for disease management. Moreover, the exact causes of many IMD remain unknown. Therefore, classical pharmacotherapy, monoclonal antibody-based or immunoglobulin replacement-based methods may produce transient or dissatisfactory results or burden unacceptable adverse effects. Therefore, search for more tailored, thus more efficient therapeutic strategies continues. Remarkably, recent advances in using cells as living drugs have revolutionized the field of medicine, offering innovative therapies for a number of diseases that were previously difficult to treat. This also holds true in the management of several types of IMD. In the current review, we aimed to summarize the cellular therapy-based approaches in IMD, marking a significant shift from traditional drug-based interventions to highly personalized precision medicine. These approaches include among others cellular therapies with hematopoietic stem cells (HSC), mesenchymal stromal cells (MSC), regulatory T cells (Tregs) or chimeric antigen receptor (CAR) T cells.

## 2 Clinical applications of cell therapies in IMD

### 2.1 Hematopoietic stem cells (HSC)

Hematopoietic stem cells, usually of the CD34^+^CD38^−^CD45RA^−^ phenotype (19), are responsible for the formation of blood and immune cells. HSC are primarily localized in the bone marrow, but after mobilization they can be also present in peripheral blood or intrinsically in umbilical cord blood. These cells have the unique ability to differentiate into all types of blood cells, which is crucial in managing of various types of diseases, including hematological cancers, solid tumors and IMD (20). HSC were primarily discovered in early 1950′s, the first allogeneic transplant was completed in 1957, while six patients were treated with intravenous infusion of marrow from a normal donor (21). Since then HSC have become crucial in regenerative medicine, treating conditions as anemia, immune system dysfunctions, and bone marrow failures (20). Indeed, HSC transplantation is currently a mainstay for treating some IMD, particularly those characterized by profound immune deficiency or, recently, highly exaggerated autoimmune dysfunctions (22). The main reason for this fact is the capability of HSC transplant to “reprogram” the immune system—it is obvious for allotransplantation, but also in autologous settings HSC transplantation can “reset” the immune system by replacing the patient's malfunctioning immune cells with new healthy cells. Therefore, transplantation of autologous HSC in an IMD patient can lead to a long-term remission by eliminating autoreactive T- and/or B cells and promoting tolerance to self-antigens, e.g., by generation of new regulatory T cell clones. Indeed, such long-term outcomes have been confirmed in patients with SLE (23), multiple sclerosis (24), systemic sclerosis (25), refractory autoimmune retinopathy (26) and other IMD (27). Nevertheless, despite multiple observations of prolonged remission and improved quality of life, especially in patients with severe, refractory forms of autoimmunity, the major limitation of autologous HSC transplantation is the requirement for myeloablation or extensive lymphodepletion. These preconditioning regimens, essential for eliminating autoreactive lymphocytes and allowing engraftment of HSC to the bone marrow make the patients exposed to potential complications, including severe infections, organ toxicity, and long-term immune suppression, which can lead to secondary cancer formation. This fact significantly restricts broader application of auto-HSC transplant as a treatment for IMD, as the risks often outweigh potential gains from this procedure. Therefore, auto-HSC transplants, especially empowered with genetic engineering of HSC, are currently considered beneficial for only a subset of individuals with autoimmunity refractory to standard treatment and/or with profound immune deficiencies (27, 28).

While discussing HSC transplantation as an anti-inflammatory cellular therapy, it is important to mention, that allogeneic HSC transplantation is burdened with a significantly high risk of Graft-vs.-Host Disease (GvHD), which, for the sake of the current review, can be definitely referred to as “immune-mediated disease”. Therefore, GvHD can be a subject for tolerogenic cellular therapies, including the ones utilizing mesenchymal stromal cells (MSC) (29) or regulatory T cells (Tregs) (30). The role of long-term observational studies in HSC transplant recipients, concerning the incidence of such complications as late cardiac events (31), gastrointestinal (32), neurological (33), and other disorders (34) is crucial for patient-oriented safety management.

### 2.2 Mesenchymal stromal cell (MSC)

MSC are present in various tissues, including bone marrow, adipose tissue, and the umbilical cord (35). They were identified and described by Friedenstein et al. (36), while conducting research on bone marrow. Since then, MSC have been extensively studied for their ability to modulate immune responses and promote tissue repair, and have been used for the treatment of poor prognosis or refractory severe AD since 1995 (37). Recognized mechanisms of MSC-mediated immunoregulatory activities include inhibition of activation and proliferation of T- and B-lymphocytes, dendritic cells, pro-inflammatory macrophages, as well as natural killer cells by arrest in the G0/G1 phase of their cell cycle (38). Furthermore, MSC cell to cell interactions are mediated by adhesion molecules, such as P-selectin, intercellular adhesion molecule-1 (ICAM-1) and vascular cell-adhesion molecule-1 (VCAM-1). It is known that these adhesion molecules trigger T-cells rolling, arrest, and then transmigration through the endothelium. MSC are able to upregulate the adhesion molecules expression and to engage T-cells to MSC (39). They have also potential to inhibit proliferation of the T-cells, in particular pro-inflammatory helper populations Th1 and Th17; and to activate Tregs (40). These properties were used in the therapeutic approaches to IMD, including RA, SLE, type 1 diabetes (T1D), multiple sclerosis and inflammatory bowel disease (41–44). For example, in 81 patients with severe and drug-refractory SLE the transplantation of allogeneic bone marrow- and umbilical cord-derived MSC was able to significantly reduce proteinuria and improve serum albumin, complement, white blood and platelet cells counts early after intravenous MSC infusion. Moreover, a significant long term decline in disease activity could be reached. The 5-year survival rate of these patients was 84%, whereas 27% achieved complete and 7% partial clinical remission (45). Beneficial effects of MSC transplantation was recently reported in SLE patients with refractory disease-related cytopenia. Significant improvement in blood cell count, along with a 43.65% reduction in disease activity index at 3-months and 72.44% at 2-years of follow-up was observed. Importantly, a 53.7% increase in Treg cells and a 54% reduction in Th17 cells were detected at one month after MSC transplantation, confirming their immunoregulatory properties (46). On the other hand, data obtained from a randomized double-blind placebo-controlled trial of allogeneic MSC transplantation for the treatment of lupus nephritis did not reveal any additional therapeutic benefit compared to standard pharmacological immunosuppression (47). The results of these studies clearly show that there is no “one size fits all” therapy for SLE. First, because it is a very heterogeneous disease and second, because SLE patients (similarly to other IMD patients) encounter numerous endogenous and environmental factors described in the section above.

### 2.3 Chimeric antigen receptor (CAR) T cells

**Figure 2:**
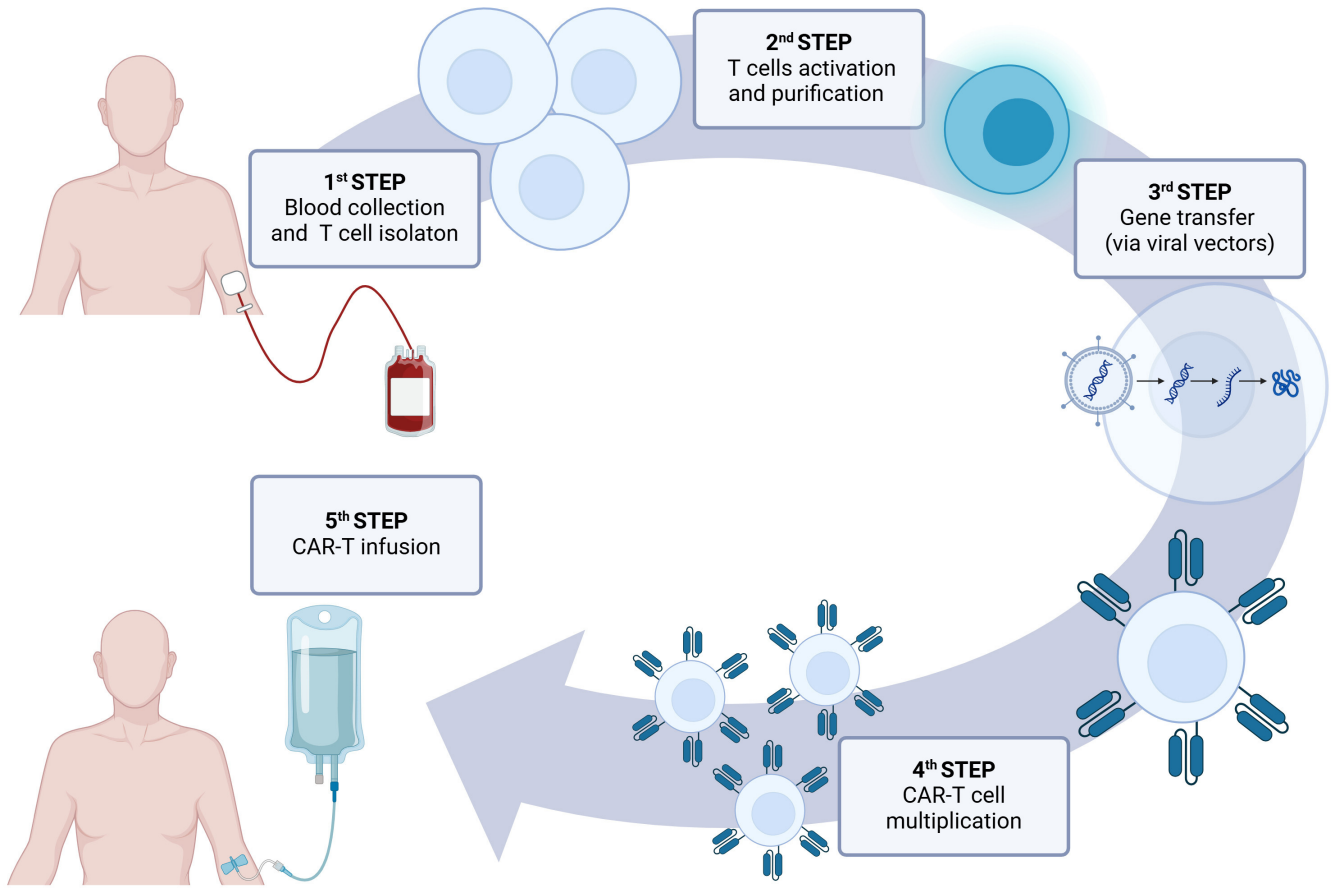
CAR-T therapy in clinical practice.

CAR-T cell therapy involves patient's T cells modification to express receptors that target and destroy specific cells (Figure 2). The term chimeric comes from the different origins of CAR components. Their extracellular antigen recognition domain is usually derived from antibodies or ligands, whereas transmembrane and intracellular activation domains are derived from T cell-specific proteins. The genetic sequence encoding CAR in a viral vector is transferred *ex-vivo* into T cells to generate CAR-T cells. After their infusion into the host they recognize the antigen, get activated, and destroy the target cell.

CAR-T cells were first mentioned in literature in 1989, by Dr. Zelig Eshhar, who pioneered their development (48). The first clinical trials were conducted in 2006 in patients with ovarian cancer (49) and metastatic renal cell carcinoma (50), these studies however did not received the Food and Drug Administration (FDA) approval. First therapy with CAR T cells (Kymriah/tisagenlecleucel), approved by FDA was launched in 2017, for the treatment of pediatric and young adult acute lymphoblastic leukemia (51). The concept of using CAR-T cells therapy in IMD is based on resetting the immune system and allowing patients to avoid immunosuppressive drugs (51). In pre-clinical setting, the use of CD19 CAR-T cells targeting B lymphocytes abrogated disease-specific B-cell autoimmunity and renal inflammation in murine SLE models (52, 53). In the treatment of human IMD, the CD19 CAR-T cells were used for the first time in 2021, in a 20-year old woman with severe SLE resistant to therapy. She tolerated well CAR-T cell infusion, which led to successful B cells depletion and CAR-T cell expansion in peripheral blood. Moreover, this approach enabled complete clinical remission and discontinuation of all immunosuppressive agents, including glucocorticoids (54). This single patient observation was recently confirmed and more profoundly analyzed in a series of five patients with treatment resistant SLE. Authors of this study reported reproducible CD19 CAR-T cells generation from their peripheral blood, despite preceding standard of care use of mycophenolate mofetil and glucocorticoids. SLE clinical manifestations remitted in all studied patients and seroconversion of anti-dsDNA, anti-nucleosomes, and anti-Sm antibodies was achieved. Moreover, humoral responses to previous vaccinations remained stable (55). The effects of CD19 CAR-T cell therapy are not limited to SLE, but can be obtained also in other IMD. One such might be a multidrug-resistant dermatomyositis, as reported in a patient who achieved immunosuppression-free clinical remission and seroconversion of anti-Jo-1 antibodies after infusion with CD19 CAR-T cells (56).

What is more, the diversity of antigen-binding domains could further expand the targeting field of CAR-T therapies (57). The B cell maturation antigen (BCMA), which is expressed on plasmablasts and plasma cells became another target. An anti-BCMA CAR-T therapy has been used in relapsed or refractory neuromyelitis optica spectrum disorders (58) and its efficacy in combination with CD19 CAR-T therapy is under investigation in SLE, Sjoegren's syndrome, necrotizing myopathies, scleroderma and vasculitis (59). Another potential target is a transmembrane glycoprotein CD7, expressed by T cells and NK cells and their precursors. Clinical trials investigating anti-CD7 CAR-T cells in refractory dermatomyositis, Still disease and inflammatory bowel disease are ongoing (59).

In addition, CAR-T cells have the potential to be used in transplantology, to prevent GvHD (60) or to enhance Graft-vs.-leukemia effect (61). That kind of therapy could also potentially be engineered to promote tolerance to the transplanted organ, reducing the need for lifelong immunosuppressive drugs or to suppress specific immune cell subsets involved in organ rejection.

Although CAR-T cells are very promising treatment tools, especially for hematological cancers, the major concern with the potential life-threatening adverse events (cytokine release syndrome and immune effector cell-associated neurotoxicity syndrome) can occur (62), therefore this strategy is a subject of further improvement (63).

### 2.4 Regulatory T cells (Tregs)

**Figure 3:**
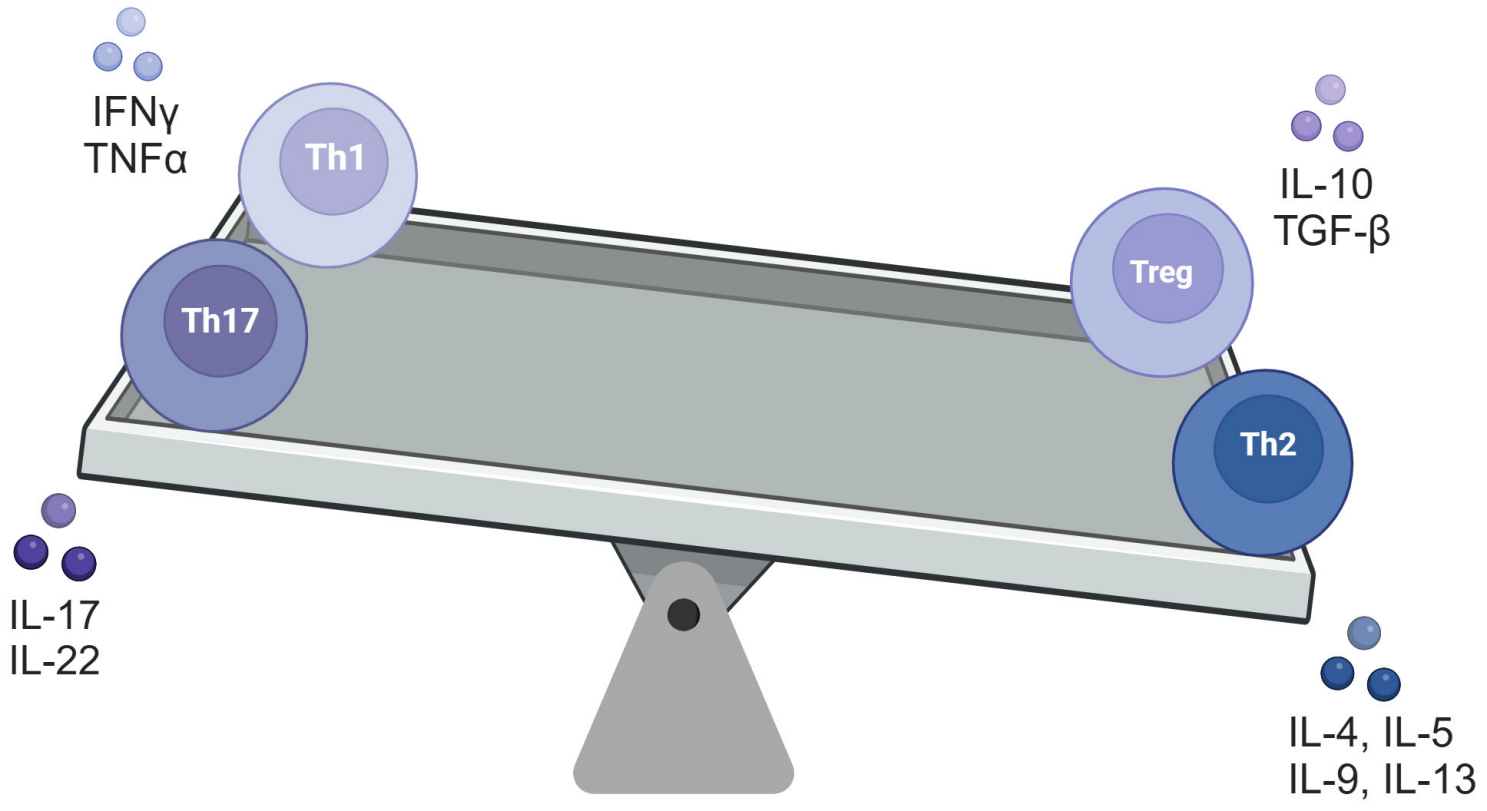
The balancing square model of four T-cell types.

Treg cell therapy are a subset of T cells which are the inflammatory response regulators, playing differential role in immune tolerance and homeostasis (64). Their clinical application involves among others: T1D, multiple sclerosis, asthma, and allergies (65–67). Treg cells were first mentioned in 1995 (68) and clinically applied in 2009 (69). It is worth to mention, that unmodified Tregs, isolated from peripheral blood, have only moderate efficacy, which can be increased by genetic modifications, such as CAR expression. This approach is thought to provide targeted CAR-Treg lymphocyte activity in target organs. The clinical and genetic engineering challenge is to prevent Tregs ability to reprogram themselves into a Th17 phenotype, with pro-inflammatory effects and its abnormal activation of the immune system. Maintaining the balance (Figure 3) between each phenotype is crucial in prevention of disease occurrence and/or progression. Recently, the efficacy of autologous polyclonal expanded Tregs were investigated in a randomized phase 2 multi-center, double-blind, clinical trial in 110 children and adolescents with new-onset T1D. The therapy was reported to be safe but it did not prevent decline in residual β cell function over 1 year compared to placebo (70). In parallel, the murine model of heart transplant, demonstrate the efficacy of CAR Treg therapy, alone or in combination with immunosuppressive agents, toward protecting vascularized grafts in fully immunocompetent recipients (71). In other preclinical study, involving mouse models, Tregs were reported to prevent severe GvHD without eliminating the potent graft-vs.-tumor effects of allo-HSC transplantation. Interestingly, such desirable immunomodulation strategy was confirmed in patients, in whom Tregs were administered ahead of conventional T cells that mediate GvHD, in some cases without any pharmacological immunosuppression (72). Also in xenogeneic transplantation the levels of Tregs early after transplant were predictive of survival. In the latter study the high levels of Tregs between days 7–17 post-transplant were associated with a GvHD-free and disease relapse-free outcome (73). Therefore, clinical trials, focusing both on efficacy and long-term safety are being conducted in various IMD, including, SLE (74), multiple sclerosis (75) or Crohn's disease (76). These trials demonstrated the safety of Treg cell therapy, while additional research is ongoing to further establish their efficacy in transplantology (NCT05987527).

### 2.5 Natural killer cells (NK)

Natural Killer cells, exhibit high cytotoxicity against tumors and virally infected cells without prior sensitization (no antigen presentation or prior exposure) (77). NK cells originating from peripheral blood, were discovered in early 1970′s (78, 79), later described as a part of the innate immune system, capable of detecting stress signals on the surface of the altered cells (80). NK cells can be also modified with CAR receptors (81). The clear advantage of CAR NK cells in comparison with CAR T cells is much lesser tendency to induce cytokine release storm, hence can be deemed safer (82). The other potential advantage is that, when CAR is directed against a T-cell specific antigen in order to eliminate autoimmune T cells, NK CAR cells would not be hampered by the fratricidal effect (83). Lastly, because NK-cells practically do not exert anti-allogenic effect, they potentially can be used as off-the-shelf therapy (84), which would dramatically lower the cost of such treatment. NK cell therapy is being actively investigated for the treatment of cancer (85, 86), infectious diseases (87), and certain IMD, such as: lupus nephritis (NCT06265220, NCT06557265), systemic sclerosis, idiopathic inflammatory myopathy (NCT06464679) and ANCA-associated vasculitis (Ntrust-2 study, Nkarta, Inc). Several clinical studies are still ongoing, to confirm the potential of NK cells as a suitable platform for IMD treatment (88, 89).

### 2.6 Dendritic cells (DC)

Dendritic cells were discovered by Ralph Steinman and Zanvil Cohn, described primary as a rare cell type in murine spleen cells with phagocytic properties (90). DCs play a critical role in the immune system as the most potent antigen-presenting cells, activating T cells and thereby initiating and regulating adaptive immune responses. DC therapy is being studied in particular for cancer treatment, with the goal of enhancing the immune system to recognize and attack cancer cells or other disease-causing agents (91). Moreover, a phase 1/2, randomized, double-blind, placebo-controlled trial of the autologous DC therapy was recently performed, enrolling participants over 16 years of age, within 1 year of T1D diagnosis. Although treatment with DC was associated with less decline in C-peptide AUC (nmol/l), compared to placebo, no clear differences in change in HbA1c and insulin dose from baseline were observed between groups (92). Thus, further studies are necessary to evaluate this therapeutic approach.

### 2.7 Induced pluripotent stem cells (iPSCs)—source of cellular therapies

iPSCs are human somatic cells that have been genetically reprogrammed to a pluripotent state, similar to embryonic stem cells. iPSCs were discovered in 2006 by Shinya Yamanaka and considered as a major breakthrough in medicine. Reprogramming technology enables the generation of patient-specific stem cells that can be used for disease modeling, drug development, screening and personalized regenerative therapies. These cells have significant potential, reducing the risk of immune rejection. iPSCs-based therapies are being investigated for several diseases, including neurodegenerative disorders, diabetes and cardiovascular disease (93). The main benefit of iPSCs is their capability to be programmed into other types of cells, including such immune cells as T cells (94), NK cells (95) and Treg like cells (96), which again rises the hope for off-the-shelf therapies.

## 3 Challenges and limitations

Despite their potential, cell therapies face several significant challenges. Although being promising, offering potentially high-effective methods for diseases with limited treatment options, they are often high-priced and such costs put these therapies inaccessible for most of the patients. It is worth to mention, that this kind of therapy usually requires individualized manufacturing procedures, involving highly specialized personnel and facilities. Many of these therapies raise important ethical issues, considering the application of gene-editing technologies and their long-term safety, particularly regarding to tumorigenesis, immune-related complications or preparation and administration protocols. The law restrictions and ethical standards vary between countries, therefore the advancements of cellular therapies may be affected.

While cellular therapies succeeded in certain areas, significant technical challenges remain. One notable setback was the recent withdrawal of Alofisel^®^ (darvadstrocel) from EU market in December 2024. An allogeneic stem cell therapy for the treatment of complex perianal fistulas in adult patients with Crohn's disease, failed to demonstrate to be more effective than placebo. Similarly, treating solid tumors with CAR-T cells has proven to be more complicated than treating hematologic malignancies. These challenges highlight broader limitations in IMD treatment, including disease heterogeneity, immune system complexity and functional stability of cell-based therapies. Therefore, ensuring both safety and efficacy of this type of therapy is a major challenge for researchers.

## 4 Conclusions and future directions

**Figure 4:**
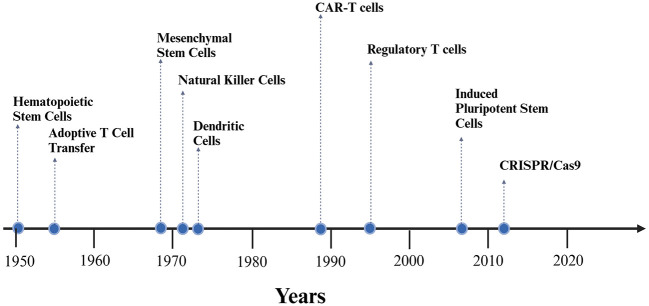
Timeline of experimental development of selected cellular therapy methods.

The field of cellular therapies is rapidly evolving (Figure 4), the improvement of safety and efficacy of these treatments is making them more affordable and accessible for patients suffering from IMD. In addition, there is significant interest in combining cellular therapies with gene editing technologies, such as CRISPR, CAST or Fanzor systems (97) to create even more precise and effective therapeutic approach.

Ongoing clinical trials are expanding the applications of cellular therapies, with promising results for diseases such as T1D (98), chronic kidney disease (99), osteoarthritis and muscular dystrophy (100), and, as mentioned above, SLE (23) and systemic sclerosis (25) (selected clinical trials are summarized in Table 2). Future therapies using allogeneic cells, which can be mass-produced and universally available, would improve accessibility and reduce the time and cost of producing patient-specific treatments.

**Table 2:** Clinical trials using cell thrapeutics in selected IMDs.

**Trial ID**	**Target disease**	**Phase**
**HSC**		
NCT04047628	Multiple sclerosis	Phase 3
NCT01174108	Aplastic anemia, paroxysmal nocturnal hemoglobinuria, myelodysplastic syndrome	Phase 2
NCT05086003	Kidney and bone marrow transplant	Phase 2
**MSC**		
NCT03917797	Systemic lupus erythematosus	Phase 2
NCT03901235	Crohn's disease	Phase 1/2
NCT04356287	Systemic sclerosis	Phase 1/2
**CAR-T**		
NCT06375993	Systemic lupus erythematosus/lupus nephritis	Phase 1
NCT05869955	Systemic lupus erythematosus, idiopathic inflammatory myopathy or systemic sclerosis	Phase 1
NCT04146051	Myasthenia gravis	Phase 2
**Treg**		
NCT05095649	Graft-vs.-host disease	Phase 1
NCT06552169	Kidney transplant	Phase 2
NCT05695521	Amyotrophic lateral sclerosis	Phase 2
**NK**		
NCT06010472	Systemic lupus erythematosus	Phase 1
NCT06377228	Lupus nephritis	Phase 1
NCT06464679	Idiopathic inflammatory myopathy, rheumatoid arthritis, systemic sclerosis	Phase 1
